# Aberrant Expression of Critical Genes during Secondary Cell Wall Biogenesis in a Cotton Mutant, Ligon Lintless-1 (*L* *i*-1)

**DOI:** 10.1155/2009/659301

**Published:** 2010-01-28

**Authors:** James J. Bolton, Khairy M. Soliman, Thea A. Wilkins, Johnie N. Jenkins

**Affiliations:** ^1^Department of Natural Resources and Environmental Sciences, Alabama University, Normal, AL 35762, USA; ^2^Plant Microbe Interactions Research Unit, USDA/ARS, Ithaca, NY 14853, USA; ^3^Department of Plant and Soil Science, Texas Tech University, Lubbock, TX 79409, USA; ^4^Crop Science Research Laboratory, USDA/ARS, Mississippi State, MS 39762, USA

## Abstract

Over ninety percent of the value of cotton comes from its fiber; however, the genetic mechanisms governing fiber development are poorly understood. Due to their biochemical and morphological diversity in fiber cells cotton fiber mutants have been useful in examining fiber development; therefore, using the Ligon Lintless (*L* *i*-1) mutant, a monogenic dominant cotton mutant with very short fibers, we employed the high throughput approaches of microarray technology and real time PCR to gain insights into what genes were critical during the secondary cell wall synthesis stage. Comparative transcriptome analysis of the normal TM-1 genotype and the near isogenic *L* *i*-1 revealed that over 100 transcripts were differentially expressed at least 2-fold during secondary wall biogenesis, although the genetic profile of the expansion phase showed no significant differences in the isolines. Of particular note, we identified three candidate gene families-expansin, sucrose synthase, and tubulin—whose expression in *L* *i*-1 deviates from normal expression patterns of its parent, TM-1. These genes may contribute to retarded growth of fibers in *L* *i*-1 since they are fiber-expressed structural and metabolic genes. This work provides more details into the mechanisms of fiber development, and suggests the *L* *i* gene is active during the later stages of fiber development.

## 1. Introduction

Cotton (*Gossypium hirsutum *L.) is a mainstay of the global economy and is prized for its excellent natural fiber properties. Cotton fibers are single-celled seed trichomes that emerge from the seed coat on the day of anthesis (dpa). Fiber length ranges from short (fuzz) to long (lint), and some genotypes can have lint as long as 50 mm. The two types of fiber possibly share common developmental pathways during early differentiation. Cotton fibers undergo near synchronous growth in discrete, yet overlapping stages: differentiation, expansion/elongation, secondary cell wall synthesis, and maturity. The rate and duration of development is linked directly to determining the fiber phenotype, and hence, fiber quality. But, despite progress from expression studies, there is no definitive link between fiber genes and fiber phenotype.

Each developmental stage is crucial for certain properties. Cell elongation is crucial for economic yield, since the quality of cotton depends largely on fiber length. After elongation, secondary cell wall thickening fills the fiber with cellulose, and this stage of biogenesis is integral for fiber fineness and strength. A combination of genetic, developmental, and physiological studies provides an extraordinary opportunity to reveal gene expression patterns associated with the fundamental process of fiber development. In order to elucidate the genetic mechanisms of fiber development, fiber mutants have been used in several recent genetic studies (Lee et al., 2006; Zhang et al., 2007) [1, 2]. Mutants in combination with microarrays provide a powerful approach to discover genes linked to key stages of fiber development, and Wu et al. (2006) [3] used six lintless or reduced fiber mutants to study the expression profiles of genes expressed during fiber initiation. Seven fiber mutants were recently mapped on cotton chromosomes, and genetic mapping of mutants is an invaluable step toward their isolation, and may provide other clues to their function (Kohel et al., 1993; Rong et al., 2005) [4, 5], but integrating them with the cotton genome and associating them with genes that affect fiber development has been slow. 

Ligon lintless (*L* *i*-1), a simply inherited, monogenic dominant mutant characterized by abnormal development that results in very short lint fiber (~2 mm long) relative to some fibers ranging between 32–34 mm in length. The shortness of the lint fibers makes them indistinguishable from the fuzz fibers. Griffee and Ligon (1929) [6] phenotypically described *L* *i*-1, but the genetic inheritance was not documented until 1972 (Kohel, 1972) [7] when the *L* *i*-1 mutant was used to demonstrate that fiber elongation and secondary wall deposition were under separate genetic controls (Kohel et al., 1974; Kohel et al., 1993) [4, 8]. The fibers of *L* *i*-1 have extensively thickened cell walls (Kohel et al., 1993) [4]. The *L* *i*-1 mutation produces a pleiotropic phenotype, as the vegetative and reproductive structures display a distorted growth pattern that is evident as early as the cotyledonary seedling stage (Kohel, 1972) [7]. *L* *i*-1 seedlings also exhibit a relatively low survival rate compared to other cotton genotypes. In addition to shortened lint fibers, the *L* *i*-1 mutation affects the rate of formation of crystalline cellulose microfibrils in both primary and secondary walls (Kohel et al., 1993) [4].

Understanding the nature of the *L* *i*-1 mutation is a key step to gaining novel insight into what genetic factors are critical for controlling fiber length, strength and cell wall characteristics crucial to important fiber traits. Although Karaca et al. (2002) [9] observed only very few changes in gene expression during fiber initiation and early stages of fiber elongation in developing *L* *i*-1 ovules, these differences could prove crucial to understanding the genetic basis of yield and fiber length. The phenotypic differences reported for the cell walls suggest that there are genes not expressed in *L* *i*-1 mutant fibers that are important to fiber cell wall biogenesis. Exploiting the *L* *i*-1 phenotype to investigate global changes in gene expression linked to aberrant crystalline cellulose synthesis and deposition holds great promise in understanding crucial aspects of cell wall structure and function in determining the fiber phenotype and spinning properties. To address this critical need, we undertook a study to discover potential genes involved in cellulose synthesis during secondary cell wall biogenesis in the *L* *i*-1 mutant using a functional genomics approach. Comparative analysis of mutant versus wild-type fibers in near-isogenic lines revealed the stage-specific expression profile of fiber genes. We identified three fiber development-related candidate genes by comparing the transcriptome profile of a normal plant with the *L* *i*-1 mutant plant in the secondary cell wall biogenesis stage. These genes, *EXPANSIN*, *tubulin*, and *SuSy* may play a crucial role in the genetic control of the *L* *i*-1 mutant. Other genes identified are discussed and may be either direct or indirect targets of the *L* *i* mutation.

## 2. Materials and Methods

### 2.1. Plant Materials

Plants of near-isogenic lines of cotton (*Gossypium hirsutum* L. cv. TM-1) and the *L* *i*-1 mutant plants were grown in the greenhouse of Alabama A&M University, Normal AL in a randomized complete block with three replicates maintained under conditions optimized for flowering (16 hours, 26 ± 2°C day; 8 hours, 20 ± 2°C night). The plants were watered and fertilized as needed. Flowers on the day of anthesis (0 days-postanthesis (dpa)) were tagged for a developmental reference point. Developing fibers of TM-1 and *L* *i*-1 were harvested at 15 and 24 dpa from first fruiting positions only. Cotton fibers from both genotypes were harvested before noon to eliminate possible diurnal effects. Fibers were separated and ground in liquid nitrogen with a mortar pestle and mortar, and stored at −80°C until RNA isolation. 

### 2.2. RNA Isolation

Twelve bolls were used to extract RNA from TM-1 and *L* *i*-1 fibers, respectively, using the modified hot-borate protocol of Wilkins and Smart [10]. There were three biological pools for each RNA sample and two independent RNA technical replicates. RNA was purified from quality-controlled total RNA using a Qiagen protocol (Qiagen, Valencia, CA). The quality of the RNA was assessed by gel electrophoresis in a formaldehyde gel and using a BioRad Genetic Analyzer. Quantification of the RNA was determined by a NanoDrop ND-1000 UV-Vis Spectrophotometer. The amount of extracted RNA varied depending on the plant genotype, the physiological age, and the amount of fiber tissue used.

### 2.3. Oligonucleotide Microarray Preparation and Analysis

Custom cotton “fiber” Agilent gene chips containing 22,000 oligoNT [60-mers], including controls, were designed and provided by Texas Tech University (Thea Wilkins) in two replicates per array in a 4 × 44 k format. Labeled mRNA probes from stage-specific ovule + fiber (15 or 24 dpa) of the mutant and TM-1 “wild-type” control plants were prepared, tested for biological variability, and hybridized to the Agilent chips using the 2Color Agilent protocol according to manufacturer's instructions (Agilent Technologies v5.5). Hybridization probes were prepared using the aminoallyl labeling method and total fiber RNA was reverse transcribed in the presence of aminoallyl-dUTP after being spiked with 2 *μ*l of test mRNA mix. Following conjugation of Cy3- or Cy5-NHS esters to reverse-transcribed cDNA with 4 dye-swap treatments and unincorporated dyes were removed from probes using QIAquick PCR Purification columns (Qiagen, Valencia, CA). Hybridization samples were prepared by incubating at 60°C for 30 minutes to fragment the RNA, followed by the addition of 2X Hybridization Buffer (Agilent Technologies, Santa Clara, CA) to stop the reaction. Samples were centrifuged at 13,000 rpm for 1 minute, placed on ice and 10 ng were loaded onto the slides and into a slide chamber. The assembled slide chamber was placed in rotisserie in a rotating (4 rpm) hybridization oven, and hybridized at 65°C for 17 hours. The slides were washed by placing them into Gene Expression Wash Buffer 1 (Agilent Technologies, Santa Clara, CA) at room temperature for 1 minute, followed by dipping them in pre-warmed (37°C) Gene Expression Buffer Wash 2 (Agilent Technologies, Santa Clara, CA) for 1 minute. Slides were immediately scanned to minimize the impact of environmental oxidants on signal intensities using Two-Color Microarray Gene Expression Analysis Agilent Scanner (Agilent Technologies, Santa Clara, CA). Spots with background and visually flagged spots with intensity less than the average were filtered. Fluorescence signals were detected through Agilent Arrays. For each signal, the mean value of replicate data points were determined in which signals lower than 1000 were omitted from further statistical analysis. Normalization and analysis were performed using GeneSpring GX 7.3 software (Agilent Technologies). The QC Reports for microarrays generated by the extraction software were analyzed, and genes with the mean normalized log_2_ intensity ≥1 were identified as differentially expressed. 

### 2.4. Genetic Analysis

Genes identified as significantly expressed were annotated by sequence similarity searches compared to the GenBank nonredundant (nr) protein database using the BLASTX program (http://blast.ncbi.nlm.nih.gov/Blast.cgi). Significant homology was determined when BLASTX e-values were less than 1e-10. Functional categories of the identified genes were assigned based on Gene Ontology (GO) annotations using online software (geneontology.org). In addition, TIGR (The Institute for Genomic Research) software (tigr.org) was used to assist in categorizing the function of significantly expressed genes.

### 2.5. Assessment of Microarray Data Using qRT-PCR

Quantitative real-time PCR (qRT-PCR) was utilized to verify the relative changes in gene expression shown during microarray analysis. Both up-regulated and down-regulated genes were identified from the microarray study, and gene-specific primers were designed from a selected number of up-regulated genes for qRT-PCR using Primer3 program (http://frodo.wi.mit.edu/). Using the same RNA used for microarray analysis, 2 *μ*g of total RNA from each sample was treated with DNase I (Applied Biosystems, Foster City, CA) for cDNA synthesis using a RETROscript Reverse Transcription for RT-PCR Kit (Applied Biosystems, Foster City, CA) for synthesis of the first-stranded cDNA. The cDNA was diluted 1 : 5 for qRT-PCR reactions carried out in a 96-wellplate in the LightCycler 480 (Roche Diagnostics, Basel, Switzerland) using SYBR Green I Master dye (Roche Diagnostics). Each reaction included 8.2 *μ*L of water, 0.4 *μ*L of forward and reverse primers, 10 *μ*l of SYBR Green I Master, and 1 *μ*L of cDNA (20 *μ*L). The amplification program was 1 cycle of 95°C for 5 minutes for preincubation, followed by 45 cycles of 95°C for 10 seconds, 58°C for 10 second, and 72°C for 15 seconds. Afterward, melting curve analysis was performed with 1 cycle at 95°C for 5 seconds, 65°C for 1 minute, and 97°C 0-s hold in acquisition mode. The amplification reaction was cooled at 40°C for 10 seconds. Each analysis contained a negative control (without cDNA template) to evaluate the overall specificity. An alpha-tubulin 2 gene (Accession# AY345604 from *Gossypium hirsutum* L.) was coamplified as an internal control. Each sample contained three replicates, and the resultant data were analyzed with the PCR efficiency correction using LightCycler 480 Relative Quantification Software (Roche Diagnostics) based on the relative standard curves describing the PCR efficiencies of the target and the reference gene.

## 3. Results and Discussion

### 3.1. RNA Isolation from 15 and 24 dpa Fibers of TM-1 and *L* *i*-1

Fiber was isolated from 15 and 24 dpa fibers of TM-1 and *L* *i*-1, and the yields of total RNA was primarily based on the specified stage of development. Average concentration of RNA from TM-1 was 4.20 *μ*g/ *μ*l and *L* *i*-1 was 3.06 *μ*g/ *μ*l (Table 1), respectively, depending on the specific date (15 or 24 dpa) of development, and RNA from 15 dpa *L* *i*-1 was less than 24 dpa. The RNA replicates were pooled prior to labeling. 

### 3.2. Analysis of Genes in TM-1 and *L* *i*-1 at 15 dpa

After examining the developmental changes versus genotypic changes (data not shown), we investigated gene expression changes in cotton at two different time points in TM-1 and *L* *i*-1. Fiber development in general requires remodeling of the fiber transcriptome [11], but microarray analysis of 15 dpa fiber revealed a similar genetic profile in the two near-isogenic lines possibly because both wild type and mutant cells are responding to developmental signals terminating elongation. At approximately 15 dpa, cotton enters a transition stage that signals the developmental switch from primary cell wall (PCW) to secondary cell wall (SCW) [12]. The expression of some genes are associated with fiber elongation, whereas others are preferentially expressed during cellulose biosynthesis, or constitutively expressed throughout fiber development. The molecular basis of the switch from elongation to cellulose synthesis remains largely unknown, although it may involve hormonal signaling, rearrangement of the cytoskeleton and oxidative burst mediated by small GTPases (Ruan 2003) [13]. We recently identified and characterized 36 preferentially expressed genes in 15-dpa fibers of a chromosomal substitution line, CS-B22sh compared to TM-1 [14], and therefore, 15 dpa was selected for this current study. Our study further validates that the primary cell wall and secondary cell wall are indeed under different genetic controls as seen in previously reported research. Elongation may be halted around 15 dpa in the ovules of *L* *i*-1, while elongation continues in TM-1 [9]. There is a high activity of 20 dpa cotton fibers in cellulose biosynthesis [15]; therefore, examining genetic and cellular events during that stage may be more plausible than at 15 dpa.

### 3.3. EXPANSIN in the Mutant Does Not Follow the Normal Genetic Expression Pattern of Its Parent, TM-1

**Figure 1:**
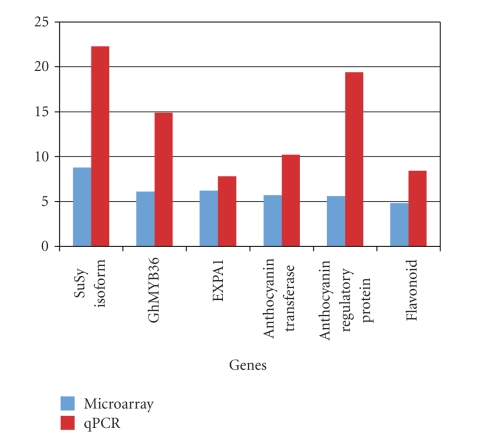
Real-time PCR of selected upregulated genes. Several of the highly expressed genes were used for validation of microarray profiles of TM-1 and *L* *i*-1.

Functional classification showed that 47.3% of the identified genes in this study were related to the cytoskeleton; 26.3% related to cell structure and organization; 15.7% related to stress response; and 10.5% related to signaling. A vast majority of the genes were of unknown function or had no similarity with accession numbers in GenBank. There was no further characterization of those genes in this study, and their percentage is not included in the above classification. Some genes had a relatively higher fold-change from qRT-PCR results than from microarray, a phenomenon reported in earlier studies by Ozturk et al. [16] and Wu et al. [14]. Overall, the qRT-PCR data of our study agreed with the data generated from the microarray analysis (Figure 1).

We observed the expression of three expansin or expansin-associated genes in our study (Table 2). Two of expansin genes, *EXPANSIN* and *EXPANSIN*-related gene, followed the normal pattern of expansin expression [17], but were down-regulated at least 3-fold in *L* *i*-1 in comparison to TM-1. However, one *EXPANSIN *gene was significantly down-regulated and is one of the most abundant transcripts in the GhTMO library [18]. This gene is notorious for its endogenous function as a cell-wall-loosening agent in Arabidopsis [19]. A second expansin gene, *EXPANSIN*-related gene, is of unknown function (Table 2), and its expression was noticeably reduced during at 24 dpa in *L* *i*-1. Generally, expansion-related genes are preferentially expressed during fiber elongation and expansion, but are known to have high levels of expression during secondary cell wall synthesis. Developmental regulation of *EXPANSIN* gene expression closely parallels that of the growth rate during the period of rapid polar elongation [11]. Most of the expansins in previous studies are classified as primary wall loosening agents and decrease after 16 dpa [20] while the minor isoforms of *EXPANSIN* are relatively high after 21 dpa [17]. An et al. [17] used transcriptome profiling to show that seven *EXPANSIN *transcripts were differentially expressed when there was parallel polar elongation during morphogenesis at early stages of fiber development, suggesting that major and minor isoforms perform discrete functions during polar elongation and lateral expansion [11]. An expansin gene whose expression was very high in our study, *EXPA1*, is a tropic response gene. This gene when over-expressed is known to cause curvatures in several organs of Arabidopsis [21], a well-defined characteristic of the fibers and leaves in the *L* *i*-1 mutant. 

### 3.4. Gamma Tubulin Complex Is Compromised in the *L* *i*-1, but Structurally Stable in TM-1

During fiber development, microtubules exhibit specific changes in orientation, organization, number length, and proximity to the plasmalemma [22, 23], and cytoskeletal changes in cotton fiber normally happen at approximately 16 to 18 dpa (Wilkins and Jernstedt, 1999) [24]. The major structural component of microtubules is alpha and beta tubulin, and most tubulins are tissue-specific. The less abundant form of tubulin, *γ*, important in the nucleation and orientation of microtubules, is also found in many higher plants [25]. While there are 6 *α*-tubulin genes in the Arabidopsis genome (Snustad et al., 1992) [26], there are approximately 30 tubulin genes expressed in a cotton fiber [27] and scientists have been able to identify nine *α*-tubulins and seven *β*-tubulin isotypes in cotton fiber cells. Expression profiling in our study showed that several isoforms of tubulin were highly up-regulated (namely tubulin beta chain and tubulin beta chain-5), while *α*-1 tubulin and *γ*-tubulin were significantly down-regulated (Table 2). Gene-specific expression of tubulins is regulated by both developmental and environmental factors. *α*-tubulin proteins increased in fiber samples throughout the developmental period between 10 and 20 dpa [27], and the increase is consistent with the increase in microtubules length and number that occurs during these stages of fiber development [23]. Our study showed there was a down-regulated expression change in mRNA in *α*-tubulin at 24 dpa fibers in *L* *i*-1 compared to TM-1 (Table 2).

Usually *α*-tubulin and *β*-tubulin self-assemble in head-to-tail arrangements to form microtubules nucleated by *γ*-tubulin and probably interacts with *δ*-tubulin, *ε*- and *η*-tubulins [28]. An isoform of *β*-tubulin, tubulin beta-9, isolated in our study was drastically down-regulated in the *L* *i*-1 mutant (Table 2). The expression of *β*-tubulin-like protein correlates positively with the elongation phase of fiber cells, and overexpression of the *β*-tubulin-like cDNA induced longitudinal growth in yeast cells [29]. We found that this gene was down-regulated in the mutant, and may affect the growth of fiber cells in *L* *i*-1. Microtubule orientation changes are due to the accumulation of both *α* and *β* tubulin isotypes [12, 14]. During later stages of development,* GhTUB* was not expressed in fiberless (*fl*) mutant ovules, but was highly expressed in wild type elongating cotton fibers. We observed that this gene was nearly defunct in *L* *i*-1 (Table 2). In previous studies, researchers established that the *GhTUB2* protein was not expressed in 0 dpa wild type ovules or in 10 dpa *fl* mutant ovules, implying it might actually be related to the elongational growth of the cotton fibers [29, 30]. 

One gene we identified that was differentially expressed was the *γ*-tubulin gene. It was down-regulated more than 2-fold in 24 dpa fibers in the mutant (Table 2). Microtubules nucleate chiefly from the *γ*-tubulin small complex and ring complex [31], and are composed of heterodimers of highly conserved *α*-and *β*-tubulins. In addition, Pastuglia et al. [32] showed that *γ*-tubulin, a gene expressed at very low levels in the *L* *i*-1 of our study, is required for the formation and organization of microtubule arrays in plant development in Arabidopsis. A recent study on keratins showed that SCP6, a component of the *γ*-tubulin ring complex, plays a role in the attachment of microtubule-organizing center (MTOCs) to intermediate filaments (IFs) [33]. Low expression of *γ*-tubulin can severely compromise the ability of microtubules to adequately assemble in the correct manner during secondary cell wall biogenesis, and this situation is seen in the *L* *i*-1 mutant. We previously identified two beta-tubulin genes differentially expressed in 15- dpa fiber of CS-B22sh compared to TM-1 [14]. In cotton fiber, high levels of *α*/*β*-tubulin proteins, including the *GhTUA9* isoform, may be required to maintain the highly dynamic polymers of microtubule arrays for rapid fiber elongation [34]. Though these tubulin isoforms are highly expressed in cotton, *γ*-tubulin, such as *TUB4*, a *γ*-tubulin involved in nucleating microtubules from both the cytoplasmic and nuclear faces of the spindle pole body, must be available to assemble the *α*- and *β*-tubulin subunits in order for the genes to be functional [35]. This may contribute to the defective fiber length in the *L* *i*-1 mutant during secondary wall biogenesis.

### 3.5. Changes in Expression Levels of SuSy Genes in *L* *i*-1 Are Observed During Secondary Cell Wall Synthesis

The differential expression of three sucrose synthase (*SuSy*) genes was observed in 24 dpa fibers of the *L* *i*-1 mutant compared to the level of fibers of its TM-1 isoline (Table 2). The expression of *SuSy* transport gene in the *L* *i*-1 mutant was significantly reduced over 4-fold (Table 2), and in an earlier study insufficient *SuSy* expression resulted in delayed initiation and distinctly shortened fiber elongation in a fuzz-like short fiber cotton mutant (Ruan and Chourey, 1998) [36]. In developing cotton fiber, sucrose synthase is localized in arrays parallel with the helical pattern of cellulose deposition, participating in the biosynthesis of cellulose [37] with sucrose being the most suitable carbon source for the production of cellulose. Scientists implicate *SuSy* as one of the potential determinants of fiber elongation as RNAi suppression in transgenic plants produces a phenotype resembling those of non-fiber-producing cotton genotype [38]. 

*SuSy* protein and mRNA are abundantly and specifically localized in initiating fibers but not in a fiberless mutant [36]. Sucrose synthase-2, *SuSy-2*, another isotype of sucrose synthase, was significantly down-regulated 4-fold in the *L* *i*-1 mutant (Table 2), and this could be a critical factor in determining the morphology/phenotype of *L* *i*-1 cotton fiber. At least half of the total *SuSy* of developing cotton fibers (*G. hirsutum*) is tightly associated with the plasma membrane [37]. Therefore, this form of *SuSy* might serve to channel carbon directly from sucrose to cellulose and/or callose synthase in or proximal to the plasma membrane [37]. Evidence for a biosynthetic role of *SuSy-2* is provided by substantially reduced starch deposition and extensive loss in fiber length and strength. Ruan and Chourey [36] showed that the expression of this same sucrose synthase gene, also seen in our study with *L* *i*-1 (Table 2), was significantly reduced in the fiberless seed (*fls*) mutant. Other microarray studies have shown that expression of *SuSy*, expansin and some transcription factors are strongly reduced in the ovule epidermis of several mutants defective in fiber initiation [3]. In addition, suppressing *SuSy* resulted in collapsed fiber initials and repression of fiber elongation in transgenic cotton plants, demonstrating the vital role of *SuSy* in fiber growth [13]. *SuS*y suppression may account for the collapsed phenotype of fiber cells (Ruan et al., 2003) [13].

In an earlier study by Ruan and Chourey [36], analyses of developing seeds 15 to 35 dpa revealed an altered temporal pattern of *SuSy* expression in the *fls* mutant relative to the TM-1 normal genotype. In our study, the expression of *SuSy *isoform remains high at 24 dpa in the *L* *i*-1 mutant as a nearly 9-fold increase was observed relative to TM-1 (Table 2 and Figure 1). This change was also reported in the *fl* mutant, and whether the altered programming of *S* *u* *S* *y* expression is the cause or result of the fiberless mutation is unknown [30, 36]. Developmentally, fiber growth parallels that of seed development, and in the *fl* mutant, high levels of *S* *u* *S* *y* were observed by Turley and Ferguson [39] in 35 dpa seeds with low amounts observed in 15 dpa. In the wild type, the authors observed a reverse pattern whereby levels of *S* *u* *S* *y* polypeptides gradually declined after 15 dpa. The *fl* mutant seeds showed a delayed program of *S* *u* *S* *y* in both RNA and protein levels. Our data suggests *SuSy* patterns were similar between the two mutants (Figure 1). In addition, large amount of sucrose in *L* *i*-1 during cell wall synthesis agrees with a previously published model [4]. They observed that the rate of crystalline cellulose formation in the primary walls of the mutant fibers correlates with the reduced rate of fiber elongation and primary wall formation. Kohel et al. [4] found a 5-fold increase in the *L* *i*-1 rate of crystalline cellulose formed per millimeter of fiber length during secondary wall formation in the mutant fibers compared to the rate in the TM-1 wild-type fibers.

## 4. Conclusion

The goal of this work was to describe differentially expressed genes at two different stages during cotton fiber development in *L* *i*-1 and TM-1 that may in turn have a more global impact on fiber growth and development. Mutants are a powerful resource for studying gene expression, and analyzing abnormal mutant plants has greatly helped in identifying and characterizing the function of specific genes. Comparison of expression of genes between the two genotypes identified over 100 transcripts that were either down-regulated or up-regulated in the fiber, even though approximately 45% were of unknown function. The cell walls of cotton have been extensively studied in both wild type and *L* *i*-1, but the genetics controlling the mechanisms of cell wall synthesis are not well understood. *L* *i*-1 gene expression has been suggested to occur in later stages or development, such as the late elongation phase [9], and this may be a plausible suggestion since our study showed no significant difference in the genetic profile in the *Li* mutant and TM-1 at 15 dpa. Molecular studies have implicated sucrose synthase [36], cytoskeleton genes like the tubulins [27, 40], and cell wall-modifying genes such as expansin (Shimizu et al., 1997) [41] as three of many potential determinants in the stages of fiber development. Several other relevant genes or their isoforms were expressed at low levels in *L* *i* versus TM-1, including genes related to energy metabolism such as ATPase synthase (Table 2) which is needed by *SuSy*, and genes associated with stress responses. In addition, the transcription factor MYB showed an expression change in *L* *i*-1 during secondary cell wall deposition. The identification and characterization of the genes that affect phenotypic expression in fiber offers valuable information for the genetic improvement of cotton fiber. 

One note to consider is that the gene controlling the *L* *i*-1 mutation may indeed be a regulatory gene, perhaps a transcription factor whose expression was not seen at 15 dpa. Even though several researchers suggest that the mutation may affect the later stages of development, the abnormalities in the phenotype are seen during the early stages of development. Therefore, pathway analysis of the genes significantly and differentially expressed in this study should be done to elucidate what steps may affect certain pathways such as flavonoid (a differentially expressed gene seen in our study), hormonal and other pathways that may be vital in cotton fiber development, especially during the early stages. The transcriptome of cotton fibers is extraordinarily complex (Hovav et al., 2008) [42], involving thousands of genes that vary in expression levels through the stages of cellular development. But understanding fiber development mechanisms will aid in increasing cotton fiber and quality, which is the final objective of cotton genetic improvement.

## Figures and Tables

**Table 1:** RNA Concentrations from *L* *i*-1 and TM-1 fiber samples at 15 and 24 dpa.

Genotype	RNA Conc. Rep. #1 (*μ*g/ *μ*L)	RNA Conc. Rep. #2 (*μ*g/ *μ*L)	RNA Conc. Rep. #3 (*μ*g/ *μ*L)	Average Conc. (*μ*g/ *μ*L)
*L* *i*-1, 15 dpa	2.9	2.9	2.8	2.9
*L* *i*-1, 24 dpa	3.1	3.0	3.1	3.1
TM-1, 15 dpa	4.1	4.2	4.2	4.2
TM-1, 24 dpa	4.3	4.3	4.3	4.3

**Table 2:** Representation of genes which are significantly upregulated or downregulated by a factor of 2X in 24 dpa *L* *i*-1 cotton fiber relative to wild-type TM-1 fiber. (Genes with unknown function or no sequence homology are omitted.).

GenBank	Description	Regulation	Fold Change	Functional Category
U73588	SuSy Isoform	Up	8.8	Cytoskeleton
AF336286	GhMYB36	Up	6.1	Cytoskeleton
AF336286	GaMYB38	Down	7.7	Cytoskeleton
DQ13871	Sucrose transport protein	Down	4.7	Cytoskeleton
AB022091	Sucrose Synthase-2	Down	4.1	Cytoskeleton
AL391142	Gamma Tubulin Component	Down	3.1	Cytoskeleton
AF521250	Alpha Tubulin	Down	2.5	Cytoskeleton
AY054693	Beta Tubulin-9	Down	2.4	Cytoskeleton
EU375992	GhTub Beta tubulin 2	Down	2.1	Cytoskeleton
NP177112	EXPA1	Up	6.2	Cell Structure/Organization
DQ02352	Expansin-related gene	Down	5.3	Cell Structure/Organization
AF043284	Expansin	Down	4.1	Cell Structure/Organization
AC152751	Putative Kinesin Light Chain	Down	3.7	Cell Structure/Organization
NM_00106	CesA7	Down	2.5	Cell Structure/Organization
AY088211	Anthocyanidin transferase	Up	5.7	Stress Response
AF336284	Anthocyanin Regulatory Protein	Up	5.6	Stress Response
AY27540	Flavonoid	Up	4.8	Stress Response
AY78110	Ethylene Transcription Factor	Down	5.9	Signaling
NM1024	Calmodulin-related protein	Down	2.7	Signaling
